# The Insane in England

**Published:** 1886-06

**Authors:** 


### THE INSANE IN ENGLAND.

The *Alienist and Neurologist*, (St. Louis, Mo.) publishes an interesting article by Dr. E. B. Nims on English Asylums, from which we make a few extracts. The whole article is too long for our crowded magazine. He visited twenty asylums in England and Scotland during the last season and remained from one to three days in each. "Three of this number were private asylums, five hospitals for the middle and upper classes, seven were county or pauper asylums, and one a criminal asylum ; the others were mixed in character. About the first thing I observed was the substantial character of the buildings, the solidity of their structure. They were plain, without ornament, as if made for use. The masses of brick and mortar are enormous. The thick walls, as seen in Bethlehem, the numerous stone and tile floors, the almost universal stone staircases, and ceilings often made of masonry, give one an idea of strength and endurance. After one has visited a considerable number of asylums he will observe that the style of building has changed from former times. Those who have seen the Bethlehem Hospital, which stands in the heart of London and which was erected in 1815, will remember the long corridors with rooms on one or both sides, also how each story is occupied, as in most American asylums. The Hamlin is similar in construction ; as likewise the Scotch asylums, as Moyanerid at Edinburgh, Gartnaval at Glasgow, and Crichton at Dumfries. In the modern asylums I observed a different order of construction, as at Berryward at Northampton, Waddy at Sheffield, and Whittingham at Preston. The day-rooms in the lower story, the lodging-rooms in the upper."

"The county asylum at Prestwick, near Manchester, is a model asylum. The main building, which is old, has been improved by bay windows and glass-covered piazzas ; the old halls have been broken into pleasant irregularity, which is a peculiarity specially liked by the superintendent. A noticeable feature is a large room which is constructed with a glass-covered roof, and which is used as an associate dining room and amusement room. The new annex, which was built for 840 patients, is made up of several buildings connected by covered corridors. Its solid walls, the interior finish of hard pine, the great amount of ornamentation in the day-rooms and the general cheerful appearance, unite to make a charming home for the pauper insane. The dry-earth closet is used here The building was erected at a cost of about £120,000. The prevalence of associate sleeping rooms in hospitals for the middle and upper classes is noticeable.

Dr. Baker has them in his new annex to the York Retreat, which was recently erected at a cost of $60,000, and which accommodates thirty patients. This building is provided with heavy plate glass windows in place of iron guards. At. Hanwell, Dr. Rayner and one attendant attended to the wants of *seven hundred patients*. At Berrywood Dr. Green and one assistant had *six hundred patients*......The ration of beer which was once universal in the asylums has been generally abolished, except for those who work and the very weak ones. The physicians have lost faith in its special value. To one who has never seen the congregate dining rooms in the large pauper asylums the first impression is peculiar. I saw them in use in three asylums. The patients marched in order to their meals ; sometimes they remained standing while grace was being said or sung. The food was served by attendants ; the patients were orderly and quiet. In one dining room there were over four hundred patients of both sexes. It seems to me that the system has many advantages. There is economy of time and saving of food in serving the meal ; waste is lessened, the wants of the patients are better attended to, any omission or neglect is much less likely to occur, and it is a relief to the halls to have the patients leave them for half an hour. The moral effect on the patients is good ; they are under a sense of restraint which increases the power of self-control and helps in what may be called the education of the insane. The idea that they are treated in a wholesale manner, that they lose their individuality, is true to a certain extent, but no more true in this than in most of the operations of a gigantic asylum."

"The amount of entertainment that is furnished at the English asylums is very noticeable : at many of them it is the custom to have field days for athletic sports, games and pic-nics. In the cricket season, which lasts most of the year, matches are played. Several of the county asylums have large, well organized bands of music, and outdoor concerts are frequent......Indoor exercises are not neglected. I saw a dance at Whittingham where six hundred patients were present. It was a well ordered and conducted exercise. Many of the asylums have fine provisions for dramatic entertainment. The airy courts which are to be seen everywhere are made attractive and pleasant and are much used."

He mentions the fact that a large amount of work is done in the extensive and elaborate grounds. In one town (Berrywood) large quantities of shoes were made by the patients. In other asylums were tailors, upholsterers, carpenters, etc. About one hundred women were at work in the laundries of each institution. A large amout of machinery is used.

"In the asylums herein mentioned there were about 15,000. I saw only *one* person that was wearing any form of mechanical restraint, and that one was a lady seventy years of age ; she had mittens on to prevent destruction of clothing. The question was always asked, 'Have you any patients in restraint ?' The answer was in every other case than the mittens, 'No ' The next inquiry was, 'Do you ever use it ?' The universal reply was, 'We should if the case warranted.' I did not find any physician who said he would not use it under any circumstances."

''Now, the question arises, what are the differences between English and American asylums? It would be supposed from some accounts that we hear, that patients in English asylums are docile and trustworthy, so that restraint is not needed, that the type of insanity is entirely different, that there is not the same violence in the onset and exacerbation of mental disease. I am sure that I saw the same furious maniacal cases that I have seen here. The patients had the same ideas and delusions, the hysterical cases were strikingly familiar, the suicidal cases were as anxious to end their lives, there was no lack of dangerous and desperate ones, yet, taken as a whole, an American would say that there is a difference in the appearance of the asylums as compared with ours, especially in the county asylums.

One thing is very evident, that is the English asylems are better managed than ours, especially the county asylums. They are not mere political places for party favorites, whether they know anything about the insane, or care anything about them ; and, we fear, ours will not be better as long as they are subject to such political gangs as are found at our large city asylums. Sometimes such terrible cases as are found out at Chicago shock the people for a time, and one set of ignoramuses give place to another of like kind. They should all be removed as far from the political machinery as possible.

